# Erythema Gyratum Repens Without an Associated Neoplasm: A Rheumatoid Twist

**DOI:** 10.7759/cureus.100593

**Published:** 2026-01-01

**Authors:** Jebisha Joseph Bella, Sudha Rangarajan, Divya Raviprakash, Leena Dennis Joseph, Adikrishnan Swaminathan

**Affiliations:** 1 Dermatology, Venereology and Leprosy, Sri Ramachandra Institute of Higher Education and Research, Chennai, IND; 2 Pathology, Sri Ramachandra Institute of Higher Education and Research, Chennai, IND

**Keywords:** erythema gyratum repens, figurate erythema, non-malignant, paraneoplastic dermatosis, rheumatoid arthritis

## Abstract

Erythema gyratum repens (EGR) is a distinctive dermatosis characterized by multiple, annular, rapidly migrating erythematous plaques with multiple concentric rings, producing a wood-grain-like pattern. Although classically described as a paraneoplastic marker, it is no longer regarded as an obligate paraneoplastic syndrome, as a growing number of cases without associated malignancy have been reported. We present a case of EGR occurring in association with rheumatoid arthritis (RA), highlighting its occurrence in a non-neoplastic context.

## Introduction

Erythema gyratum repens (EGR) represents a rare variant of figurate erythema distinguished by swiftly expanding, ring-shaped plaques that advance outward in layered patterns, imparting a characteristic wood-grain morphology. Historically, EGR has been regarded as a cutaneous sign considered highly suggestive of an underlying visceral cancer, especially tumors arising from the lung, breast, esophagus, and stomach [1]. Nevertheless, eruptions have been increasingly recognized in non-neoplastic contexts, including pityriasis rubra pilaris, psoriasis, ichthyotic disorders, infectious etiologies, drug exposures, and a spectrum of connective tissue disorders [2,3]. The occurrence of EGR in rheumatoid arthritis (RA) appears to be exceedingly uncommon, with only isolated cases reported in the literature [4]. This report represents an additional documented instance of EGR in association with RA in the absence of any detectable hidden neoplasm, underscoring the importance of acknowledging autoimmune contributions while maintaining rigorous evaluation for occult malignancy.

## Case presentation

A 67-year-old postmenopausal female, a known case of RA on oral methotrexate, presented to the dermatology outpatient department with a three-year history of gradually progressive, pruritic, dark, raised lesions over the abdomen and lower limbs. There was no history of weight loss, loss of appetite, dysphagia, altered bowel habits, breathlessness, or postmenopausal bleeding.

**Figure 1:**
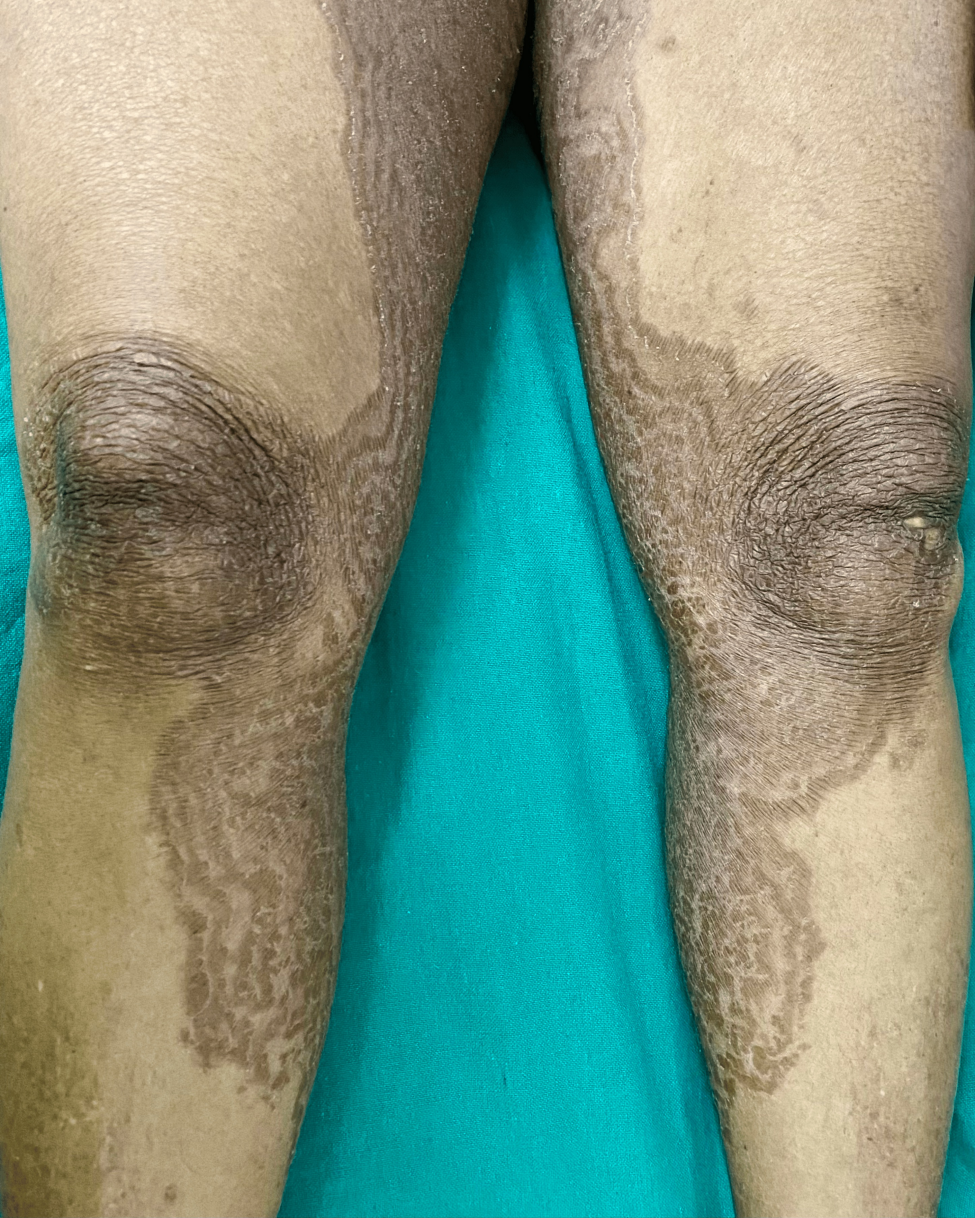
Well-defined concentric erythematous to hyperpigmented, scaly gyrate plaques with rippled margins, producing a wood-grain-like pattern, over the medial aspects of both thighs and legs.

**Figure 2:**
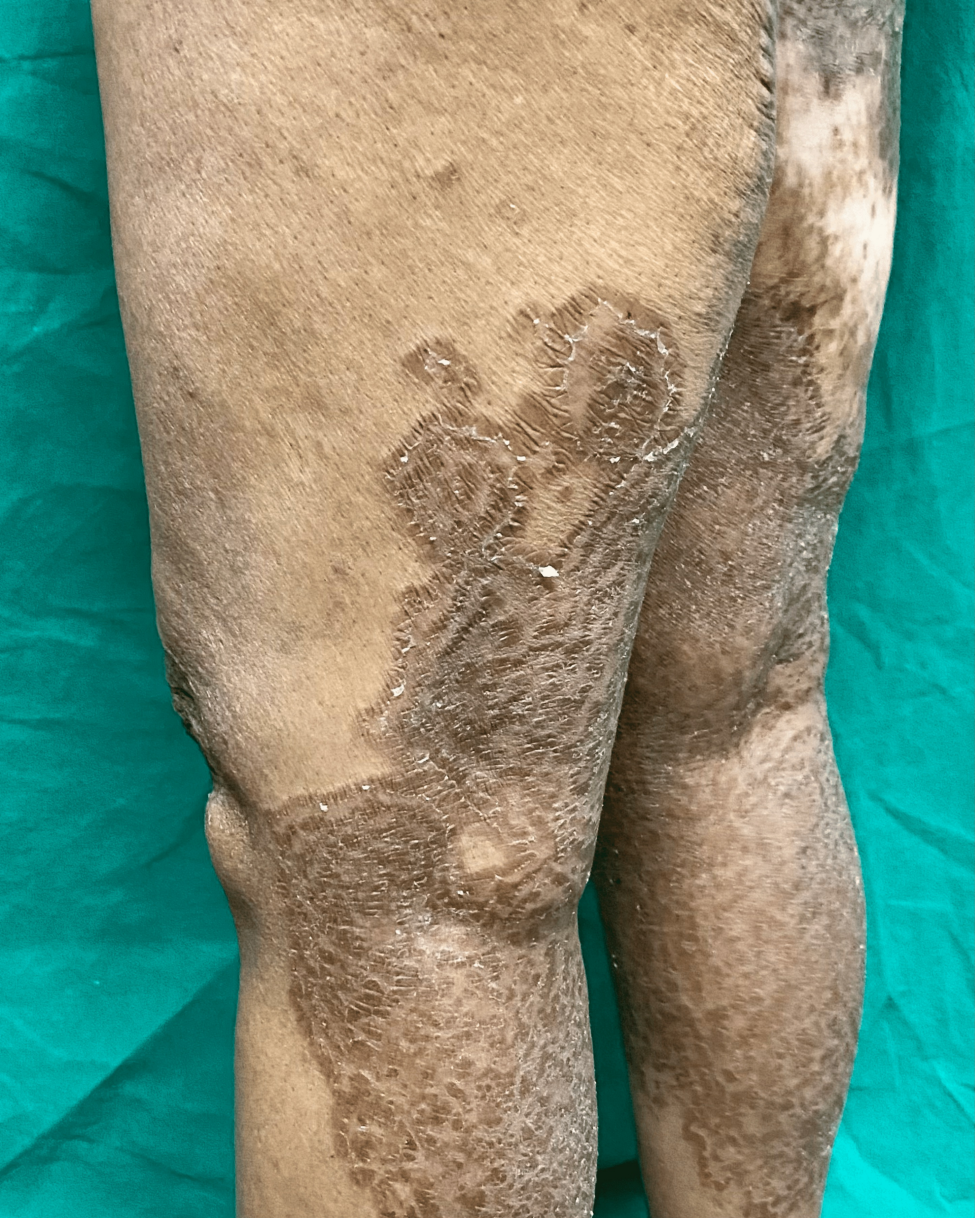
Lesions extending to posterior thighs and legs bilaterally.

**Figure 3:**
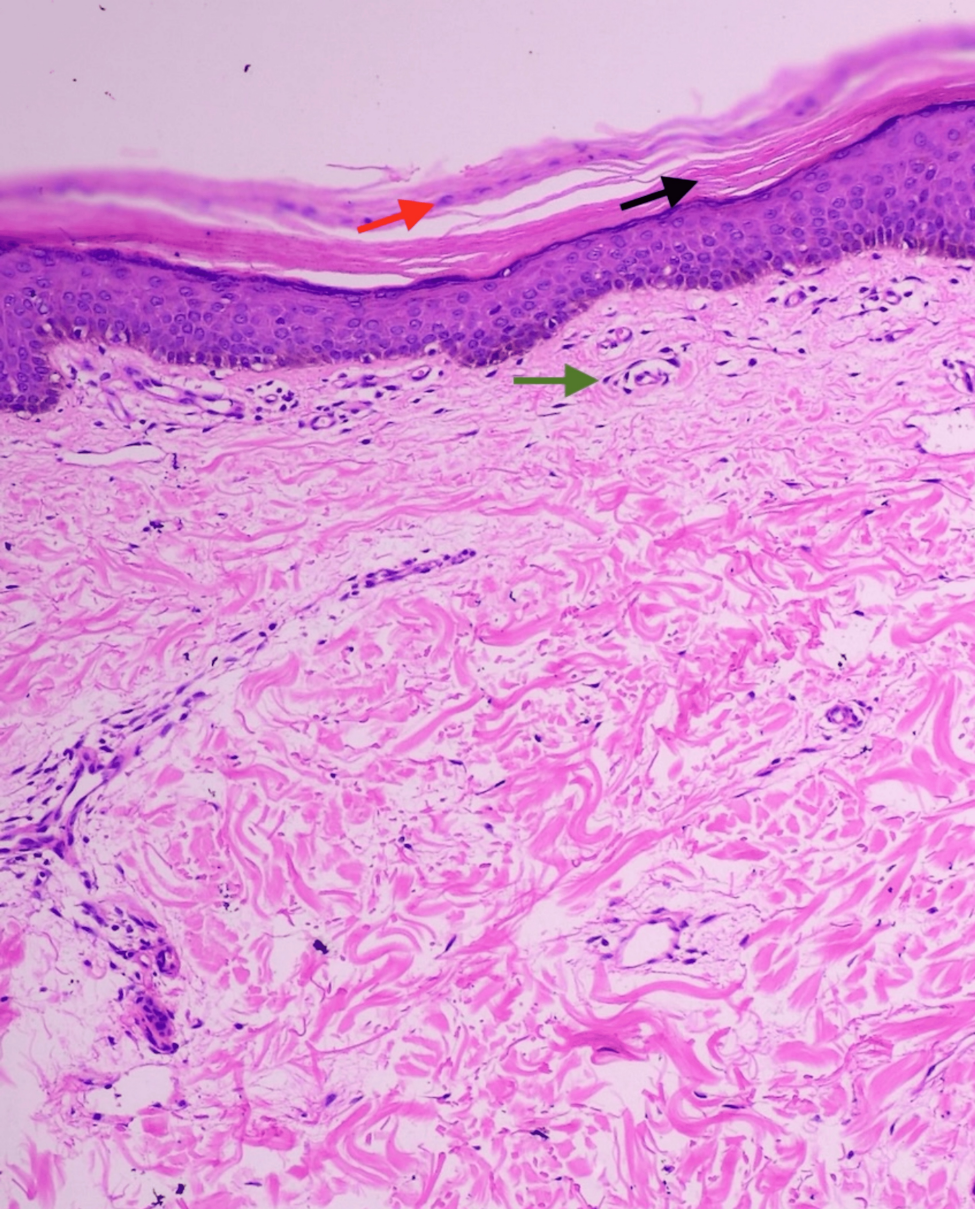
Histopathological examination (hematoxylin and eosin stain, 40x) showing basket-weave hyperkeratosis (black arrow), focal parakeratosis (red arrow), and superficial perivascular lymphocytic infiltrate (green arrow).

Examination revealed well-defined concentric erythematous to hyperpigmented scaly plaques with wave-like borders, resembling wood-grain, over the abdomen, bilateral medial aspect of thighs and legs (Figure 1), and bilateral posterior thighs and legs (Figure 2). The differential diagnoses considered were EGR and tinea imbricata. The potassium hydroxide (KOH) mount did not reveal any fungal elements. Histopathology showed basket-weave hyperkeratosis, focal parakeratosis, and superficial perivascular lymphocytic infiltrate, which was consistent with EGR (Figure 3).

Extensive malignancy workup with Pap smear, chest radiography, abdominal ultrasonography, and whole-body positron emission tomography-computed tomography (PET-CT) was negative. She was continued on oral methotrexate and started on symptomatic treatment with oral antihistamines and topical corticosteroids. Lesions demonstrated only minimal improvement over six months. The patient has subsequently been followed for two years without evidence of malignancy and continues regular follow-up with annual malignancy screening.

## Discussion

EGR, first described by Gammel in 1952, is an uncommon dermatologic entity, notable for its rapid centrifugal progression and concentric, figurate pattern [5]. Although rare, its recognition is clinically important due to historical associations with internal malignancy. In population-based reviews, the majority of cases, approximately 80%, have been linked to neoplasia, most frequently involving the lung, followed by esophageal, breast, gastric, renal, and hematological cancers [1,6]. Notably, the eruption may precede, coincide with, or follow the diagnosis of the underlying neoplasm [6,7]. The non-malignant causes include drug-induced EGR-like eruptions, inflammatory skin disorders like psoriasis and pityriasis rubra pilaris, pulmonary tuberculosis, hypereosinophilic syndrome, ichthyotic dermatoses, and immune-mediated connective tissue diseases [2,3].

The exact pathogenesis of EGR remains unclear; however, current evidence supports an immunologically driven mechanism. It has been proposed that the eruption arises from a cross-reactive immune response between tumor-derived antigens and structurally similar cutaneous antigens. Supporting this hypothesis, deposits of immunoglobulin G (IgG) and complement component 3 (C3) have been demonstrated along the basement membrane zone in both lesional and non-lesional skin, as well as within associated neoplastic tissue. Another proposed mechanism posits that specific tumors may release polypeptide molecules that bind to epidermal antigens, modifying them sufficiently to elicit an immune response [7,8]. Chronic inflammatory states, as seen in autoimmune diseases, could therefore provide a permissive environment for EGR, explaining their rare occurrence in disorders such as RA.

The eruption typically presents as multiple annular or polycyclic, erythematous plaques forming wood-grain-like patterns with sharply demarcated borders and scales at their edges. The lesions demonstrate a centrifugal spread at a remarkable pace, often up to 1 centimeter per day, which is considered a hallmark feature of the condition. Pruritus is common and may be severe, contributing to significant patient discomfort. Cutaneous involvement in EGR typically favors the trunk and proximal extremities while sparing the face, palms, and soles [6].

Histopathology in EGR is supportive but non-specific. Findings often include epidermal hyperkeratosis, parakeratosis, mild spongiosis, and superficial perivascular lymphocytic infiltrate [9]. Consequently, clinical evaluation remains the cornerstone of diagnosis.

Therapeutic intervention in EGR is largely directed toward detecting and treating the underlying neoplasm. Non-paraneoplastic EGR appears to follow a more indolent course compared with paraneoplastic cases and may respond partially to symptomatic therapy [10]. Therapeutic attempts have involved both topical and oral corticosteroids, vitamin A derivatives, and immunosuppressive agents like azathioprine, but responses are generally limited [1,11].

## Conclusions

This case highlights an unusual instance of EGR arising without an associated malignancy in a patient with established RA. Reporting such cases is critical to expand the understanding of EGR’s broader etiologic spectrum and its potential relationship with immune dysregulation. Despite the characteristic clinical and histopathological features, a comprehensive diagnostic workup failed to uncover any occult malignancy. Given the traditionally strong association of this dermatosis with internal cancers, ongoing clinical surveillance remains essential. This case, therefore, also emphasizes the need for long-term malignancy follow-up even in the setting of a known autoimmune disease and when initial investigations are unremarkable.
